# Immune Adjuvant Effect of Molecularly-defined Toll-Like Receptor Ligands

**DOI:** 10.3390/vaccines2020323

**Published:** 2014-04-25

**Authors:** Deana N. Toussi, Paola Massari

**Affiliations:** Section of Infectious Diseases, Department of Medicine, Boston University School of Medicine, Boston, MA 02118, USA; E-Mail: Deana.Toussi@bmc.org

**Keywords:** TLR, vaccine adjuvant, immune response, microbial pathogens, cancer

## Abstract

Vaccine efficacy is optimized by addition of immune adjuvants. However, although adjuvants have been used for over a century, to date, only few adjuvants are approved for human use, mostly aimed at improving vaccine efficacy and antigen-specific protective antibody production. The mechanism of action of immune adjuvants is diverse, depending on their chemical and molecular nature, ranging from non-specific effects (*i.e.*, antigen depot at the immunization site) to specific activation of immune cells leading to improved host innate and adaptive responses. Although the detailed molecular mechanism of action of many adjuvants is still elusive, the discovery of Toll-like receptors (TLRs) has provided new critical information on immunostimulatory effect of numerous bacterial components that engage TLRs. These ligands have been shown to improve both the quality and the quantity of host adaptive immune responses when used in vaccine formulations targeted to infectious diseases and cancer that require both humoral and cell-mediated immunity. The potential of such TLR adjuvants in improving the design and the outcomes of several vaccines is continuously evolving, as new agonists are discovered and tested in experimental and clinical models of vaccination. In this review, a summary of the recent progress in development of TLR adjuvants is presented.

## 1. Introduction

The main goal of vaccination is to induce immunologic protection from infectious diseases of bacterial, viral and parasitic origin. Host immune responses to a given vaccine antigen can be greatly enhanced by simultaneous administration of an immune adjuvant. Adjuvants are exogenous substances that have a wide variety of nature and origin, ranging from mineral salts, oil and water-based emulsions, polymers, microparticles, liposomes, saponins, microbial products and even cytokines [1,2,3].

Despite the importance of their influence on the immune response, the mechanisms of action by which most adjuvants potentiate innate and adaptive immunity have only recently begun to be understood. Adjuvants are generally categorized into delivery systems and immunostimulatory adjuvants. Delivery systems, particulate adjuvants and emulsions including alum [4], water-in-oil and oil-in-water emulsions (*i.e.*, Complete Freund’s Adjuvant (CFA) [5] or MF59 [6]) are thought to generate an antigen depot at the site of injection, which is then slowly released over time (although other factors have been described to contribute to the effect of alum and MF59, for example [7,8]). This process leads to enhanced antigen uptake and presentation by antigen presenting cells (APCs) and induction of high antigen-specific antibody titers. The second category of vaccine adjuvants, immunostimulatory substances, enhances immune responses via a direct effect on immune cell activation and function. These adjuvants induce: (1) upregulation of surface expression levels of the major histocompatibility complexes I and II (MHC I and MHC II) on APCs and enhanced antigen presentation to the T-cell receptor (TCR) (Signal 1); (2) APC maturation/activation and increased surface expression of co-stimulatory molecules (CD40, CD80, CD86) needed for proper activation of naïve T cells (Signal 2); (3) direct and indirect immunomodulation and differentiation of T lymphocytes; 4) recruitment of immune cells at the site of injection and migration to the draining lymph nodes [9]. In addition, both categories of adjuvants induce immune cell responses mediated by inflammatory mediators (*i.e.*, cytokines and chemokines (signal 3)) [9] and surface receptors/adhesion molecules. The convergence of the events elicited by immune adjuvants leads to enhanced adaptive immune responses and subsequent immune protection, particularly through the activation of dendritic cells (DC) and T cells [10]. 

It has also been established that activation of APCs occurs via specific recognition of microbial products, a step that has been defined as Signal 0 [9], and is required for innate immune responses that guide T helper cells towards Th1-, Th2- and Th17-type differentiation. Th1-type responses are defined by the pro-inflammatory cytokines IL-12, IFN-γ and TNF-α, by high levels of IgG2a/b (or IgG2c), IgG3 and IgA in mice, and IgG1, IgG3 and IgA in humans, cell-mediated immunity (CMI) via both CD4^+^ T cells and CD8^+^ cytotoxic T cells (CTLs) (although the latter also require antigen presentation via MHC class I). Th2-type responses are defined by IL-4, IL-5, IL-6, IL-10 and IL-13 and CD4^+^ T cell-dependent B cell-mediated humoral immunity via induction of IgG1 and IgE/ IgA in mice or IgG4 and IgE in humans [11]. Dysregulation of Th1-type responses to self-antigens or the commensal flora leads to tissue destruction and chronic inflammation, while dysregulation of Th2-type responses is implicated in allergy and asthma. Recently, Th17-type responses, characterized by IL-17 and IL-23 [12] have been described to modulate neutrophil recruitment [13], and B and T cell functions, including those of regulatory T cells (Treg) [14], thus playing a role in vaccine development [15]. Therefore, inclusion of adjuvants in vaccine formulations is important for both stimulation of innate immunity and induction of improved antigen-specific adaptive responses. 

Various adjuvants have been shown to mediate different types of adaptive immune responses. For example, alum (the first USDA-licensed adjuvant approved for use in humans in the US and present in over 80% of the licensed human vaccines) stimulates Th2-type responses and strong antigen-specific IgG1 and IgE antibody production, but it does not induce CD8^+^ T-cell immunity and may even inhibit Th1-type immune responses [16]. By contrast, adjuvants such as QS-21 (a saponin from the Soap bark tree *Quillaja saponaria* in an oil-in-water emulsion), MF59 or Freund’s complete adjuvant (CFA) induce preferentially Th1-skewed responses, or a mixed Th1/Th17-type and Th1/Th2-type immunity [3].

In the early 1990s, the potential for a number of bacterial and viral components to act as immune adjuvants has been elucidated by their ability to interact with specific host cell receptors that recognize microbial molecular patterns, the Toll-like receptor family (TLRs) [17]. The role of TLRs in regulation of host innate and adaptive immune responses has been explained by their ability to induce activation of immune cell signaling. In B cells, TLR signaling induces up-regulation of surface markers involved in antigen up-take (MHC I and MHC II) and in cross-talk with T cells (CD40, CD80, CD86), ultimately enhancing antigen-specific antibody production when TLR ligands are used combined with antigens in the context of vaccination. In addition, TLR signaling also plays a role in induction of B- and T-cell memory. In APCs, including B cell, DCs and macrophages, TLR signaling also results in enhanced secretion of both pro- and anti-inflammatory mediators that drive development of T helper cell subsets into Th1-, Th2- or Th17-type, depending on the type of APC involved [18]. Generally, signaling via TLR3, TLR4, TLR7, TLR8 and TLR9 promotes Th1-type immune responses while signaling via TLR2 (along with TLR1 or TLR6) and TLR5 favors Th2-type immune responses [19,20]. TLR ligands also influence Treg development [21]. A direct influence of TLR signaling on Treg development has been shown, due to expression of functional TLRs on these cells, as well as an indirect effect, due to Treg interaction with TLR-activated APCs [22]. TLR signaling can lead to either Tregs functional activation or suppression, depending on the TLR ligand type and effect on antagonistic induction of Th17 cells [21]. This aspect is particularly relevant for cancer, autoimmunity and chronic inflammation, due to the effects of Th17-type cytokines (IL-17A, IL-17F and IL-22) [21,23]. This review discusses the mechanisms of action of TLR agonists with vaccine adjuvant properties and highlights their potential use to improve vaccination against infectious diseases and cancer.

## 2. TLR Signaling Mechanism and Pathways

Toll-like receptors (TLRs) comprise members of a family of related trans-membrane proteins that recognize microbial and viral products. TLRs have been categorized as pattern recognition receptors (PRRs) that recognize ligands from pathogenic microorganisms (the “pathogen-associated molecular patterns” (PAMPs) [24]), from commensal organisms (the “commensal-associated molecular patterns” (CAMPs) [25]) and endogenous ligands deriving from damaged cells (the “danger-associated molecular patterns” (DAMPs)) [26].

**Figure 1:**
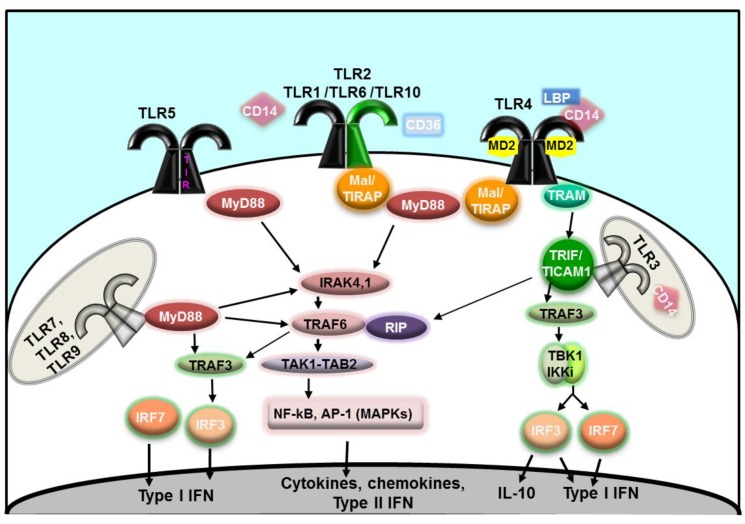
Schematic cartoon of Toll-like receptor (TLR) signaling [17,24,27,28,29,30]. Extracellular TLR homodimers (TLR4 and TLR5) are represented in black; heterodimers of TLR2 and TLR1, TLR6 or TLR10 are indicated in black/green. Intracellular homodimers (TLR3, TLR7, TLR8 and TLR9) are indicated in gray.

The structure of TLRs is that of horse-shoe shaped proteins composed of three domains: an extracellular or cytoplasmic leucine-rich repeat (LRR) domain which mediates ligand recognition, a single trans-membrane domain, and an intra-cytoplasmic domain, the TIR domain, homologous to the corresponding intracellular domain of the IL-1 receptor (IL-1R) Toll/IL-1R [17]. In humans, 10 TLRs have been identified so far. TLR1, TLR2, TLR4, TLR5, TLR6 and TLR10 are surface-expressed and recognize extracellular ligands and microorganisms, while TLR3, TLR7, TLR8 and TLR9 are situated on endosomal membranes within the cell and are engaged by intracellular ligands and microrganisms [17]. Ligand binding and TLR homo- or heterodimerization brings the TIR domains of adjacent TLRs together, providing a conformational change necessary to trigger signaling. Binding of additional adaptor proteins is also essential for intracellular cascades. Adaptor proteins include the myeloid differentiation factor 88 (MyD88) [27], the MyD88 adaptor-like protein (Mal/TIRAP), the TIR domain-containing adaptor protein inducing interferon-β (TRIF/TICAM) and the TRIF-related adaptor molecule (TRAM) [28,29] (Figure 1). Negative regulators of TLR function have also been identified and include the Toll-interacting protein (Tollip), IRAK-M, the α- and HEAT-Armadillo-motif-containing protein (SARM) and the B cell adaptor for PI3K (BCAP) [30].

All TLRs except TLR3 require MyD88 recruitment to the TIR domain for signaling activity [27]. TLR2 and TLR4 also require the cooperation of the adaptor protein Mal/TIRAP. In the MyD88-dependent signaling pathway, activation of IRAK4 and IRAK1 (members of the IL-1R-associated protein kinases (IRAKs) [31]) is followed by that of TRAF6 (tumor necrosis factor receptor-associated factor 6 [32]) and RIP (receptor interacting protein [33]), with subsequent signal transfer to a complex made of TAK1 (TGF-β-activated kinase 1), TAB1, TAB2 and TAB3 (TAK1-binding proteins 1, 2 or 3) and, ultimately, activation of NF-κB and, through members of the mitogen-activated protein kinase (MAPK) family (ERK, JNK, p38), activation of AP-1 [34]. The MyD88-dependent TLR signaling pathway leads to host cell responses involved in cell survival/proliferation and immune pathways culminating with immune cell activation, induction of inflammatory mediators and antimicrobial products. Signaling through TLR7, TRL8 and TLR9 also activates a parallel MyD88-dependent cascade through IRF7 (interferon regulatory factor 7 [35]), followed by TRAF6, IRAK4 and TRAF3 activation and leading to type I interferons (IFN) production (Figure 1). 

TLR3 signaling activates a MyD88-independent pathway via TRIF and TRAF3 [36], leading to activation of IRF3 and resulting in secretion of IFN-β and IL-10 (Figure 1). The TLR3-TRIF signaling also drives activation of MyD88-dependent pathway downstream components, TRAF6 and RIP1, converging on activation of NF-κB and AP-1. Similar to TLR3 signaling, TLR4 can also induce MyD88-independent signaling, although TRIF recruitment is not direct but requires prior activation of TRAM. Downstream cell activation via both TRAF6/RIP1, as well as TRAF3-IRF3, provides an amplification of the cytokine repertoire (Figure 1). 

## 3. TLR Adjuvants with a Preferential Th1-bias

### 3.1. TLR3-Dependent Adjuvants

TLR3 (CD283) is expressed in endosomal compartments in myeloid dendritic cells (mDCs) and, weakly, in monocyte-derived macrophages [37] and it recognizes viral double-stranded RNA (dsRNA) that is produced during viral replication in infected cells, with the potential contribution of CD14 [38]. A synthetic analog of dsRNA, Poly I:C (polyriboinosinic:polyribocytidylic acid) [39], has similar immunostimulatory properties, inducing TLR3 activation via the TRIF/TRAM pathway and secretion of inflammatory cytokines and IFN-β (Figure 1). However, Poly I:C also interacts with other receptors, such as the retinoic acid-inducible gene I (RIG-I), melanoma differentiation-associated gene 5 (MDA-5) and double stranded RNA-dependent protein kinase [40], which may possibly influence its adjuvant activity. Nevertheless, since TLR3 ligands favor strong cellular Th1-type immune responses, they have been tested as adjuvants in vaccines against viral infections. Activation of dendritic cells by TLR3 agonists not only contributes to induction of innate and adaptive immune responses against microbial pathogens, but also favors NK cell activation and killing of tumor cells by stimulating anti-tumor CD8^+^ T cells [41]. 

Poly I:C has been used as an adjuvant in various experimental vaccine models. However, the major draw-backs of Poly I:C are its low stability and toxic side-effects. Pre-clinical studies carried out in primates have shown that Poly I:C is easily degraded by serum nucleases, with a consequent reduction of IFN secretion and anti-tumor activity [42]. Unfortunately, increasing the dosage of Poly I:C has not proven a successful strategy, as this TLR3 ligand is not well-tolerated. Thus, several derivatives of Poly I:C have been synthetized and tested for safety and adjuvanticity, such as Poly ICLC and Poly I:C12U (Table 1).

Poly I:C favors antigen cross-presentation to primed CD8^+^ T cells, due to TLR3-dependent increased MHC class I expression and type I IFN secretion, as well as development of antigen-specific cytotoxic T cell clones [43]. Poly I:C inclusion in an HIV vaccine based on purified recombinant gp120 antigen has shown development of MHC class I-restricted CD8^+^ cells *in vivo* [44]; in another HIV vaccine strategy, addition of Poly I:C (and CpG DNA) to DNA encoding for a Gag antigen/anti-DEC205 antibody fusion protein has shown improved mucosal antigen presentation on MHC class I molecules and enhanced CD4^+^ T cell-mediated immunity [45]. Several studies in experimental animal models support the efficacy of vaccine formulations containing TLR3-based adjuvants [46]. In the quest for vaccines against cancer, the use of Poly I:C has shown enhancement of tumor specific T cell responses [47]. For example, in an ovarian cancer vaccine, Poly I:C enhances DC maturation and IL-12 secretion [48].

Poly I:CLC (Hiltonol®) is a synthetic double-stranded polyriboinosinic-polyribocytidylic acid stabilized with poly-L-lysine carboxymethyl cellulose and is less sensitive to serum-degradation [49]. Poly ICLC induces high IFN-γ secretion and enhances CTL responses and antigen-specific antibody titers, although mild to severe side effects have been reported when it is used at high doses [50,51]. Poly ICLC is currently being tested in clinical trials for both tumors and infectious diseases.

**Table 1:** Examples of TLR adjuvants, disease models tested in experimental and clinical trials, and human vaccines. Disease models and corresponding vaccines are shown in bold.

TLR	Ligand	Disease Models	Human Vaccine
*TLR3*	Poly I:C,	HIV [44,45,52], HPV [51],	
	Poly I:CLC (*Hiltonol*),	Influenza [46,53],	
	Poly I:C12U (*Ampligen*),	Cancer [47,48,50,54]	
	Poly I:C + CAF01 *(CAF05)*		
*TLR4*	Monophosphoryl Lipid A (MPL),	**HBV** [55,56,57,58,59],	***Supervax/Fendrix*** [55,58,59],
	RC-529 *(Ribi*),	*Leishmania* [60], TB [61,62],	
	MPL/QS-21/liposomes *(AS01),*	VZV [63], Malaria [64,65,66,67],	
	MPL/QS-21/oil-in-water	HIV [68,69], **HPV** [70,71,72,73],	***Cervarix*** [72,73],
	emulsion *(AS02)*,	HSV [74], EBV [75],	
	MPL + Alum *(AS04)*,	**Melanoma** [76,77],	***Melacine*** [76],
	MPL + DETOX, AGPs,	**Cancer** [78,79,80]	***Stimuvax*** [78],
	GLE-(SE), E6020, OM-174,	RSV [81], *L. monocytogenes* [81],	***Theratope*** [79]
		Influenza [81,82]	
*TLR7 TLR8*	Imiquimod (*R-837*), Resiquimod (*R-848*)	**HPV** [83,84], **Molluscum** [84],	***Aldara*** [83,84,85,86, 87]
		**Cancer** [83,84,85,86], **Melanoma** [87], HIV [88], HSV [88], ***Leishmania*** [89]	
*TLR9*	CpG ODN, CpG ODN + MPL/QS21 (*AS15*)	Malaria [90,91,92],	
		**Influenza** [93],	***Fluarix*** [93],
		**HBV** [94,95,96,97], Anthrax [98], HPV [99],	***Engerix-B*** [96],
		Cancer [99,100,101,102,103],	***Heplisav*** [97]
		Melanoma [100,102,104,105],	
*TLR2/TLR1 TLR2/TLR6*	Lipoproteins, MALP-2, Pam_2_CSK_4_, Pam_3_CSK_4_; non-lipidated ligands: porins (*Neisseriae*, *F. nucleatum*, *Chlamydia*, *Salmonella*, *Shigella*), toxins* (E. coli* LT-IIa-B(5)/IIb-B(5))	enterohemorragic *E. coli* [106,107],	
		**Lyme** **Disease** [108,109]	***LYMErix*** [109]
		Malaria [110,111], **HBV** [112,113]	***Theradigm-*HBV** [112]
		HIV [114,115], *Chlamydia* [116], *Salmonella* [117], *Neisseriae* [118,119], Influenza [120,121], Helminths [122], *F. tularensis* [123]




*TLR5*	Flagellin	*Y. pestis* [124], West Nile virus [125], *L. monoctyogenes* [126], Malaria [127,128], Dental Caries [129], Cancer [130,131], HPV [131], ***Influenza*** [132,133,134,135,136,137,138]	***VAX128*** [134], ***VAX125*** [135], ***VAX102*** [137], ***STF2.4xMe*** [138]

Poly I:C12U (Ampligen®), a synthetic Poly I:C containing mismatched bases (uracil and guanine), is also immunostimulatory while less toxic than Poly I:C and Poly I:CLC. Intranasal immunization with Poly I:C12U as an adjuvant in a hemagglutinin (HA)-based H5N1 influenza vaccine induces higher levels of protective, specific mucosal IgA and systemic IgG responses than the corresponding adjuvant-free vaccine [53]. In phase II and III clinical trials for HIV vaccines, and in phase I and II cancer vaccine studies, Poly I:C12U has been deemed safe to use and induces maturation of mDCs, secretion of IL-12 and inhibition of IL-10, enhances antigen-specific CTL responses and Th1-type CD4^+^ T cell responses [54,139]. 

Poly I:C has also been combined with a cationic adjuvant formulation, CAF01, a liposome-based adjuvant composed of dimethyldioctadeclammonium and trehalose-6,6-dibehenate (DDA/TDB) [140]. The combined Poly I:C-CAF01 adjuvant is called CAF05. Through the effect of Poly I:C, CAF05 enhances CD8^+^ T cell responses and, through the effect of CAF01, induces a long lasting antigen depot. CAF05 favors Th1-type and Th17-type immunity and antibody responses in animal models of bacterial, viral and parasitic infections, and also has an effect on reducing tumor growth rates [52,141]. 

### 3.2. TLR4-Dependent Adjuvants

TLR4 (CD284) is expressed by the majority of circulating immune cells but its mature form has been characterized in macrophages and mDCs [37,142]. TLR4 signals via both the MyD88-dependent and the (MyD88-independent) TRIF-dependent pathway, leading to a robust IL-12 production, secretion of type I IFNs and a strong Th1-type cellular and humoral immune response (Figure 1). 

A number of TLR4 ligands has been described, with lipopolysaccharide (LPS) being the first bacterial product shown to interact with this receptor [143]. Other TLR4 agonists include a variety of components from fungi, viruses and parasites and endogenous ligands [144,145,146,147]. The TLR4/LPS molecular interaction has been elucidated in detail [148]. LPS has a hydrophilic polysaccharide component and a hydrophobic lipid A, composed of polyacylated diglucosamine lipids. The lipid A interacts with the TLR4 accessory molecule, lipid A binding protein (LBP) [149], followed by formation of a complex with CD14 (soluble or cell wall-anchored via glycosyl-phosphatidylinositol (GPI)), which is then presented to TLR4 and the myeloid differentiation protein 2 (MD-2) [150]. 

LPS, along with its molecular derivatives, has been tested in numerous vaccine clinical trials (Table 1). However, despite its strong immunostimulatory effect, an intrinsic toxicity severely limits its use in humans. A detoxified form of LPS, the monophosphoryl lipid A (MPLA) from *Salmonella minnesota* R595, was developed by Ribi [151]. MPLA retains a potent immunostimulatory activity *in vitro* and *in vivo* while lacking toxicity, and is used in a number of complex adjuvants broadly referred to as Ribi adjuvant systems (RAS). For example, synthetic MPL RC-529 (Ribi.529) is used in the human hepatitis B virus (HBV) recombinant antigen vaccine, SupervaxTM [55]. MPLA triggers both the MyD88-dependent and TRAM/TRIF-dependent pathway, although an apparent preferential bias towards signaling via the TRIF-dependent pathway has been reported [152]. Induction of a strong protective Th1-biased immunity and secretion of pro-inflammatory mediators (*i.e.*, TNF-α) by MPLA has been shown for *Leishmania* and TB vaccine formulations, as well as induction of anti-inflammatory mediators (*i.e.*, IL-10) [61,70]. 

MPLA has been combined with a variety of other adjuvants, such as QS21 and liposomes (AS01, GlaxoSmithKline (GSK) Vaccines), QS21 and an oil-in-water emulsion (AS02 (GSK)), and alum (AS04 (GSK)) [153] (Table 1). Due to the effect of MPLA, AS01, AS02 and AS04 all induce TLR4-dependent NF-κB activity and cytokine secretion, maturation and trafficking of DCs and monocytes to the draining lymph nodes and antigen-specific T cell activation (although AS04 does not directly activate B or CD4^+^ T lymphocytes) [154].

In experimental and clinical trials, AS01 has been shown to induce Th1-type immunity, improve CD8^+^ T-cell responses and high antibody titers, for example to TB, varicella zoster virus (VZV), HIV antigens and to the malaria antigen RTS,S (a *P. falciparum* surface protein fused to the HBV surface antigen (HBsAg)) [63,64,68]. AS02, which has also been tested with HBV, HIV, TB and malaria antigens, elicits a more balanced Th1/Th2 immunity, with lower lymphoproliferative responses and a shorter-lived protection than AS01 [62,65,68]. By contrast, AS02 induces higher CD8^+^ cytolytic T cell responses than AS04, the MPLA/alum adjuvant. In the AS04 adjuvant, the MPLA/antigen complex is stabilized by the presence of alum, which also favors formation of antigen depot. AS04-containing vaccines have been tested against viral pathogens, including HBV [57], HPV [70,71], herpes simplex virus (HSV) [74] and Epstein-Barr virus (EBV) [75] showing improved protective responses than the corresponding alum-alone containing vaccines. AS04 is part of the HBV vaccine, FENDrix® [58], which has been safely and successfully used in healthy adults and in specific high-risk patients [55,59]. The AS04-containing vaccine against HPV, Cervarix®, is prophylactically used against cervical cancer and is also well tolerated [72,73]. In addition, MPLA-containing adjuvants have been used in cancer vaccine formulations, for example with the MUC1 antigen against prostate cancer or non-small cell lung cancer (NSCLC) [78] (Stimuvax®), or in combination with the adjuvant DETOX® (an oil-droplet complex that contains purified *Mycobacterium phlei* cell wall skeleton products (CWS) [76]) in a melanoma vaccine (Melacine®). DETOX is also used with the MUC1/ KLH antigen complex (Theratope) in breast and ovarian cancer treatment [79]. AS02 has also been used with the recombinant melanoma-associated antigen 3 (MAGE-A3) in cancer vaccine approaches, with some success [77]. The AS01, AS02 and AS04-adjuvanted vaccines are considered safe [66,67,69]. 

Based on the success of AS04, and on the different chemical composition of MPLA species, the adjuvant effect of other synthetic lipid A mimetics with different length and degree/type of fatty acid acylation has been examined (Table 1). For example, aminoalkyl glucosaminide 4-phosphates (AGPs), tested against *L. monocytogenes*, influenza and RSV [81], the E6020 synthetic molecule [155], and the RC-529 (Ribi.529) molecule, structurally similar to the hexa-acyl component of MPL® [65]. These synthetic lipid A mimetics are considered safe. Another synthetic lipid A derivative, glycopyranosyl lipid adjuvant (GLA) has been used in combination with squalene (SE, an oil-in-water emulsion), and shown to induce strong Th1-type responses, enhance antigen-specific responses and have a good safety profile [82]. Lastly, the lipid A derivative, OM-174 from *E. coli*, has also been tested for its adjuvant effect [80].

### 3.3. TLR7- and TLR8-Dependent Adjuvants

TLR7 and TLR8 (CD288) are expressed in neutrophils, monocytes, macrophages, eosinophils and B cells (TLR7), plasmacytoid DCs (pDCs) (TLR7), NK cells and T cells (TLR8) and Langerhans cells [156]. Similar to TLR3, TLR7 and TLR8 have an intracellular localization within endosomal compartments in the cells that express these receptors. Engagement of TLR7 and TLR8 leads to signaling through MyD88 ⁄Mal, NF-κB and IRF7 activation and secretion of proinflammatory cytokines, chemokines and other mediators (Figure 1). In DCs, TLR7/TLR8 activation leads to cell maturation/activation, expression of co-stimulatory molecules (CD80, CD86 and CD40), enhanced antigen presentation and secretion of Th1-type pro-inflammatory cytokines (IFN-α, TNF-α and IL-12). pDCs respond to TLR7 activation by secreting IFN-α while mDCs respond to TLR8 activation by producing IL-12 [157]. Both TLR7 and TLR8 induce Langerhans cell differentiation and migration from the skin to the lymph nodes. Signaling via TLR7 induces secretion of Ig, IL-6 and TNF-α by B cells [158] and IFN-γ by NK cells [159]. TLR8 signaling induces T cell proliferation, IFN-γ, IL-2 and IL-10 production, memory T cell activation and also reduces CD4^+^ Treg-mediated immunosuppression [160]. 

The ligands for TLR7 and TLR8 include single stranded (ss) RNA enriched for poly-U or poly-GU sequences [161], synthetic imidazoquinolinamines, such as imiquimod (R-837) and resiquimod (R-848) [162] and guanosine analogues, such as loxoribine. While TLR7 or TLR8 agonists are not approved as vaccine adjuvant components, imiquimod and resiquimod have undergone extensive clinical testing in a 5% cream formulation (AldaraTM) for topical treatment of HPV-induced warts, actinic keratoses, basal cell and squamous cell carcinoma, lentigo maligna and molluscum contagiosum [83,84] (Table 1). Both compounds induce strong local secretion of IFN-α, TNF-α, IL-6 and IL-12, as well as cytotoxic T-cell responses. Topical application of imiquimod-containing formulations has also been tested in prostate cancer vaccines, favoring development of specific CTL responses and antibodies [85]. In a melanoma trial, systemic co-administration of imiquimod, a melanoma peptide vaccine and Flt-3 ligand (a DC activator) resulted in enhanced peptide immunogenicity and recruitment of both mDCs and pDCs in the treated areas. Imiquimod topical application also favors development of a T cell-dependent response to intradermal injection of the melanoma antigen, NY-ESO-1 [87]. In various tumor animal models, the combination of DNA-based vaccines and imiquimod treatment has been successful in reducing tumor onset, increasing CTL responses and IgG2a antibody production [86]. In pre-clinical studies on HSV and HIV, antigen-specific T cell responses and antibody secretion are enhanced by imiquimod [88], and in *Leishmania* infections, macrophage-dependent bacterial killing and resolution of cutaneous lesions have been reported following use of imiquimod [89]. Unfortunately, systemic administration of imiquimod is highly toxic and studies on TLR7/TLR8 adjuvant safety and efficacy are limited by the unresponsiveness of mice to TLR8 agonists for human use [161].

### 3.4. TLR9-Dependent Adjuvants

In humans, TLR9 (CD289) is expressed by immune cells in intracellular endosomal compartments and its role is particularly relevant in B cells and pDCs [163]. TLR9 signals through the MyD88 pathway via IRAK and TRAF-6 without the contribution of Mal (Figure 1), leading to production of Th1-type pro-inflammatory cytokines (IL-1, IL-6, IL-12, IL-18, TNF-α and IFN-γ), up-regulation of CD80, CD86, CD40 and MHC molecules expression, increased antigen processing/presentation and CD8^+^ T cell responses [164,165]. In particular IL-12 and type I IFNs induced in pDCs via TLR9 drive a strong Th1-type immunity and CD8^+^ CTL cytotoxicity, while TLR9-dependent B cell activation leads to increased antigen-specific humoral responses and IgG class switching [166,167]. 

The ligands for TLR9 are bacterial and viral DNA that contains unmethylated CpG motifs and synthetic oligodeoxynucleotides (ODN) expressing CpG motifs [168]. The synthetic TLR9 ligands retain the immunostimulatory activity of bacterial DNA and are divided in three major classes, based on their structure, biological properties and ability to activate immune cells *in vitro* [169,170]. Multiple CpG motifs on a phosphorothioate backbone are classified as “K” type ODN (also called “B” type), which are strong inducers of B cell activation, pDCs and monocyte maturation. “D” type ODN (also called “A” type), have a mixed phosphodiester/phosphothioate backbone containing a single CpG motif flanked by palindromic sequences and 3'- and 5'-end poly-G tails that allow formation of concatamers. These CpG ODN activate NK cells. The third category, “C” type ODN, is structurally and functionally similar to both “K” type and “D” type ODN, with both phosphorothioate nucleotides and palindromic CpG motifs, and induce activation of both B cells and pDCs and production of IFN-α. 

Numerous pre-clinical and clinical studies have been carried out with TLR9 adjuvants (Table 1) [171,172]. The adjuvant activity of “K” type ODN has been explored in vaccine models against malaria [90,91], HBV [94,95], influenza [93] and anthrax [98]. CpG ODN induces a strong specific antibody response to the malarial Apical Membrane Antigen 1 (AMA1) and to the merozoite surface protein 142 (MSP142) (both poorly immunogenic vaccine candidates) [92]. In the case of HBV, the B type CpG ODN, CPG 7909, enhances specific, long-term antibody responses to the Engerix-B® vaccine (recombinant HBsAg vaccine absorbed on alum (Alhydrogel)), as compared to Engerix-B alone [96]. Another CpG ODN, the 1018 immunostimulatory sequence (ISS), has shown to improve the efficacy of the HBV vaccine Heplisav®, with only minor local side effects [97]. By contrast, inclusion of CpG 7909 in the influenza vaccines Fluarix® is considered less substantial, although it enhances IFN-γ secretion and is well tolerated, which is advantageous for reducing the vaccine dosage [93]. 

CpG-ODN is also used in anti-cancer vaccines and immunotherapy, due to its ability to induce high numbers of tumor-specific cytotoxic CD8^+^ T cells when co-administered with HPV and melanoma tumor antigens [99,100]. In vaccine trials with the synthetic tumor peptide MART1 (melanoma-associated antigen recognized by T cells 1) (Melan-A) and with the NY-ESO-1 peptide antigen, addition of CpG ODN enhances antigen-specific CD8^+^ T cell responses [101,104]. CpG 7909 has only shown partial success when used in a MAGE-A3 protein-based vaccine, which has been improved by addition of MPL and QS21 in a liposomal formulation to CpG 7909, the AS15 adjuvant. The AS15-adjuvanted vaccine induces an increased MAGE-A3 delivery to APCs and enhances T-cell immunogenicity [105] (Table 1). However, despite a good safety profile of CpG 7909, intra-tumoral injection of this TLR9 adjuvant has shown scarce results on tumor growth in melanoma and basal cell carcinoma models [102]. Similarly, evaluation of CpG 7909 administration during chemotherapy for NSCLC treatment, or combined with GM-CSF and the tumor antigen, hTERT (human telomerase reverse transcriptase), has not shown a great success rate [103].

## 4. TLR Adjuvants with a Preferential Th2-bias

### 4.1. TLR2-Dependent Adjuvants

TLR2 (CD282) expression is relatively ubiquitous in immune cells and is found on the surface of neutrophils, macrophages, monocytes, basophils, T cells, B cells, NK cells and immature DCs [37]. TLR2 dimerizes with either TLR1 or TLR6 and also utilizes other accessory molecules, such as CD36, CD14 and LBP [173,174,175,176]. TLR2-dependent signaling proceeds through the Mal/TIRAP and MyD88-dependent pathway, inducing activation of NF-κB and MAPKs pathways leading to immune cell activation, survival/proliferation, secretion of inflammatory mediators and expression of co-stimulatory molecules (CD80, CD86 and CD40) (Figure 1). 

TLR2 interacts with structurally diverse ligands. Natural and synthetic lipopeptides and lipoproteins that signal via TLR2 include *M. fermentans* macrophage-activating lipopeptide (MALP-2), a TLR2/TLR6 ligand [173], the syntetic triacylated lipoprotein, Pam3CSK4, a TLR2/TLR1 ligand [174] and the diacylated lipoprotein, Pam2CSK4, a TLR2/TLR6 ligand [177]. TLR2 also binds peptidoglycans (PG) [178], glycosylphosphatidyl-inositol-anchored structures from gram positive bacteria (lipoteichoic acid, LTA), lipo-arabinomannan from *Mycobacteria* and lipomannas from *M. tuberculosis* [179]) and other cell wall components (*i.e.*, β-glucans [180] and zymosan [181]), as well as viral products [182] and some bacterial LPS types (reviewed in the TLR4 section). Endogenous ligands and DAMPs [183] and several lipid-free bacterial proteins have also been described as TLR2 ligands, including porins, toxins, fimbriae [184] and the PPE18 protein from *M. tuberculosis* [185]. The molecular and structural details of TLR2 interaction with some of its ligands have been elucidated, while other TLR2/agonist complexes are currently being explored [186,187,188]. 

The adjuvanticity of TLR2 agonists has been characterized as predominantly Th2-biased. The most extensively studied TLR2 adjuvants include MALP-2, Pam3CSK4, *Neisseria* PorB and *E. coli* LT-IIa-B(5) and LT-IIb-B(5) (Table 1). A large majority of studies conducted with these adjuvants have been carried out in experimental animal models, and most are not yet approved for routine administration in humans. In experimental and pre-clinical studies, TLR2 adjuvants support DC and B cell responses and T cell activation, including that of antigen-specific CD8^+^ T cell (CTL), although at relatively modest levels as compared to other TLR adjuvants [189]. Treg functions can also be influenced by TLR2 activation; for example, TLR2/TLR1 signaling may mediate protective mucosal Th17-type responses to pathogens and Treg cells expansion, while TLR2/TLR6 signaling may promote tolerogenic dendritic cells and Treg responses [190]. 

MALP-2 has been used as an adjuvant in a number of experimental immunization studies with prototype antigens, such as ovalbumin (OVA), but also in disease models. Intranasal administration of MALP-2 in a vaccine model against enterohemorragic *E. coli* enhances secretion of antigen-specific serum IgG and mucosal IgA, IFN-γ, IL-2 and IL-4 [106]. The synthetic derivative of MALP-2, BPP (*S*-[2,3-bispalmitoyiloxy-(2R)-propyl]-*R*-cysteinyl-amido-monomethoxyl polyethylene glycol), also enhances secretion of antigen-specific antibodies and cytokines (TNF-α, IL-10 and MIP-1β), and favors antigen cross-presentation by DCs to CD8^+^ T cells and cytotoxic T-cell response [191]. 

Lipoproteins have been also used in experimental and clinical vaccine studies. In a vaccine against Lyme disease, immunization with the *B. burgdorferi* outer surface lipoprotein A (OspA) with alum has shown enhanced secretion of protective antibodies against a C-terminus epitope of OspA [108]. This Lyme disease vaccine, LYMErix®, was licensed in 1998 after showing a good safety profile in clinical trials. However, potential concerns regarding skewing of Treg responses toward a Th17 phenotype and induction of autoimmune disease have stopped its commercialization [109]. Pam3CSK4 has been used in an anti-malarial vaccine containing several *P. falciparum* circumsporozoite protein (CSP) B cell epitopes and a universal T cell epitope, demonstrating induction of relatively high titers of peptide-specific IgG and IgG1, IgG3 and IgG4 antibody subclasses in immunized volunteers [110]. Lipid-containing TLR2 adjuvants can be easily conjugated to vaccine antigens, and even antigens themselves can be modified by addition of a lipid-core peptide for TLR2 interaction and direct activation of immune cells [192]. Such a strategy has been employed in a vaccine containing an HBV core antigen CTL peptide and a helper T lymphocyte (HTL) peptide conjugated with a palmitic acid at the N-terminus (Theradigm-HBV) [112]. In a phase I trial, this vaccine has shown higher immunogenicity than the un-palmitoylated vaccine and induction of long-term, dose-dependent, HBV-specific CTL responses in healthy subjects [113]. Phase I and II trials of an HIV-1 lipopeptide-based vaccine have shown similar long-lived, antigen-specific IgGs and specific CTL responses [114]. 

Porins from *Neisseriae*, *F. nucleatum*, *Chlamydia*, *Shigella*, *Haemophilus* and *Salmonella* have been examined in numerous experimental immunization and pathogen challenge models. Generally, bacterial porins have a rather conserved, trimeric structure consisting of monomers with a high content of β-barrel structure. In the bacterial membrane, porins mediate passage of ions and solutes for organism survival [193]. Purified porins can be formed into stable native preparations, called proteosomes, and are recognized by TLR2 on the surface of immune cells. Porins from *Neisseriae*, *F. nucleatum* and *Chlamydia* have a TLR2/TLR1-dependent adjuvant activity [194,195,196], while the adjuvanticity of *Shigella* porin and *Salmonella* OmpS2 is mediated by TLR2/TLR6 signaling [117,197]. Remarkably, *Neisseria*, *F. nucleatum*, *Chlamydia* and *Salmonella* porins induce Th2-type skewed immune responses [116,173,195] while *Shigella* porin appears to favor Th1-type responses [198]. The TLR2-dependent porin effects include APC activation/proliferation, increased surface expression of CD80 (*Shigella*, *Salmonella*), CD86 (*Neisseria*, *F. nucleatum*, *Chlamydia*), CD40 and MHC II molecules and induction of antigen-specific IgM, IgG and IgA antibodies [118,119,196,199,200]. Neisserial porin proteosomes have been tested as adjuvants in mucosal and systemic vaccinations against different pathogens in both experimental and clinical models without side effects or toxicity [111,120,121,122,123,201]. 

Toxins from *Enterobacteriacee,* divided in type I (the cholera toxin (CT) and the *E. coli* heat-labile enterotoxin I (LT-I)) and type II (the *E. coli* LT-IIa, LT-IIb and LT-IIc) [202,203,204], are also potent mucosal immune adjuvants, although their clinical development is severely compromised by their high toxicity in humans [205]. Enterotoxins are oligomeric proteins composed of an A subunit, responsible for the enzymatic activity of the toxin, and a pentameric B subunit (B5), which mediates binding to ganglioside receptors on host cells (*i.e.*, GM1, GD1b and GD1a, GQ1 and GT1). Genetically detoxified type I toxin A and B subunits, including the LTK63, LTR192G, LTR72 and LTH44A molecules, have been tested in experimental vaccine models against bacterial, viral and parasitic infections, in cancer vaccines and in clinical trials [107,115,206,207], but their safety remains under scrutiny [208]. Besides binding to ganglioside receptors, the B subunit of type II LT (LT-IIa-B(5) and LT-IIb-B(5)) also binds to TLR2 [209,210], via regions that are normally masked by the A subunit in the whole holotoxin [209]. Interaction of LT-IIa-B(5) and LT-IIb-B(5) with the TLR2/TLR1 heterodimer is facilitated by binding to the GD1a ganglioside and leads to APC activation, secretion of high levels of antigen-specific systemic and salivary IgG and IgA antibodies, memory B cell development, secretion of cytokines (with high IL-4 and low IL-12), repression of Treg development/function and increased Th1, Th17 and especially Th2-type responses [204,210,211,212]. Additionally, LT-IIa induces CD8^+^ T cell apoptosis, thereby reducing IFN-γ secretion and further influencing Th-type immunity [213]. The TLR2-dependent functions are retained by non-toxic mutants of the LT B(5) subunit, such as LT-IIb(T13I), which fail to bind their ganglioside receptors [214,215]. 

### 4.2. TLR5-Dependent Adjuvants

TLR5 is expressed on the surface of neutrophils, monocytes, mDCs, Langerhans cells, T cells and NK cells [163,216,217]. Signaling through TLR5 via the MyD88 ⁄Mal pathway leads to a strong induction of NF-κB activation and a preferential Th2-type immunity (although a Th1 component can be also present) [218,219] (Figure 1). The ligand for TLR5 is bacterial flagellin and the TLR5-binding region is located in a conserved region of flagellin, the D1 portion [220,221,222,223,224]. A number of experimental models have demonstrated that flagellin, in both soluble monomeric and polymeric forms, has an immune adjuvant effect and induces DC maturation/activation with subsequent up-regulation of CD80, CD83, CD86 and MHC class II, secretion of IL-10 and TNF-α by monocytes and IFN-γ and α-defensins by NK cells, T cell proliferation/activation and antigen-specific CTL responses [224,225]. Although likely not through a direct effect on B cells, but more to a general TLR5-dependent enhancement of APC functions, immunization with flagellin-containing vaccines also leads to enhanced secretion of antigen-specific IgG and local IgA responses [222]. For example, addition of flagellin in an intranasal influenza vaccine in mice has shown enhancement of immune response as compared to the vaccine without flagellin [133,134]. Similar effects have been shown in experimental models of vaccination against *Y. pestis* [124], West Nile virus [125] and *L. monocytogenes* [216] (Table 1).

A major advantage of this TLR5-dependent adjuvant is its use in fusion proteins with recombinant antigens, which has shown induction of superior immune responses as compared to simultaneous co-administration of flagellin and antigens [19,127,226]. This approach has been used in experimental and clinical trials of vaccines against influenza, using a flagellin/hemagglutinin-based vaccine (VAX125, VAX128) [134,135,136] or a flagellin/matrix protein 2 ectodomain (M2e) vaccine (VAX102) [137,138], and in vaccines against malaria [127,128], vaccinia virus [227], *P. aeruginosa* [228] and even against dental caries [129]. Although the safety of flagellin-based vaccines is still being evaluated in clinical trials, no major local or systemic side effects have been reported so far. 

In addition to its use as adjuvant for vaccines against infectious diseases, flagellin has also been used in cancer treatment, where NF-kB and transcriptional regulation of mediators of apoptosis are induced by flagellin via TLR5 signaling. It is thought that reduction of apoptotic cell death may be beneficial for the consequences of radiation treatment in normal tissues. In experimental irradiation studies in rodents and primates, improved survival rates have been observed following vaccination with flagellin-derived polypeptide (CBLB502) [130]. In other studies on potential anti-cancer strategies, flagellin has also shown an enhanced generation of tumor-specific CD8^+^ T cell immune responses [131].

## 5. Conclusions

The ultimate goal of vaccination is to generate protection against diseases. Such protective immunity requires induction of different host responses that are elicited by using vaccine formulations containing appropriate antigens and adjuvants. Adjuvants are important components of vaccines and can influence the outcomes of vaccination, particularly by directing host immune responses towards different T helper cell immunity and enhancing both the quality and the quantity of immune response against the antigens. However, major concerns in vaccine adjuvants development include their safety and efficiency. Even though vaccine design is still rather empirical, recent advances in immunology research have expanded our understanding of the mechanisms of action of various adjuvants and greatly improved the chances for successful development of safe and effective interventions to prevent and treat a number of human diseases through modulation of host immune responses. The discovery of TLRs and their role in modulation of innate and adaptive immunity has led to exploitation of their ligands as immune modulators, due to their ability to induce specific immune cell activation and influence host adaptive immunity. The advantage of TLR adjuvants is not only in their ability to preferentially induce Th1 or Th2 responses and development of CD4^+^ or CD8^+^ T-cells, but also to modulate B cell activation and enhance antibody secretion to otherwise poorly immunogenic antigens, improving both quality and quantity of specific antibody production. Furthermore, TLR adjuvants appear suitable for enhancing mucosal immunity, an area that is gravely underdeveloped in the current human vaccine strategies. 

As discussed here, experimental models and clinical trials evaluating TLR agonists as immune adjuvants have identified valuable molecules for use in vaccines against infectious diseases, allergies and cancer immunotherapy (Table 1). In particular, TLR3, TLR4 and TLR9 agonists have been shown to improve a number of vaccines, for example against HBV, influenza, malaria and anthrax, as well as some types of cancer. TLR7/TLR8 agonists are less developed as adjuvants but are already used with success in topical cancer immunotherapy. The efficacy of vaccine formulations containing traditional adjuvants has also been reported to be synergistically improved by the addition of TLR agonists. It is likely that the known TLR ligands described here and potentially other novel TLR ligands with adjuvant effect, could be introduced in human vaccine formulations worldwide in the near future as both stand-alone adjuvant systems or in combination with existing non-TLR adjuvants in the design of next-generation vaccines [19,126,229]. 
